# Plasma proteomic signatures of enteric permeability among hospitalized and community children in Kenya and Pakistan

**DOI:** 10.1016/j.isci.2023.107294

**Published:** 2023-07-11

**Authors:** Kirkby D. Tickell, Donna M. Denno, Ali Saleem, Zaubina Kazi, Benson O. Singa, Catherine Achieng, Charles Mutinda, Barbra A. Richardson, Kristjana H. Ásbjörnsdóttir, Stephen E. Hawes, James A. Berkley, Judd L. Walson

**Affiliations:** 1 Department of Global Health, University of Washington, Seattle, WA, USA; 2 The Childhood Acute Illness & Nutrition (CHAIN) Network, Nairobi, Kenya; 3 Aga Khan University, Karachi, Pakistan; 4 Kenya Medical Research Institute, Nairobi, Kenya; 5 Department of Biostatistics, University of Washington, Seattle, WA, USA; 6 Department of Epidemiology, University of Iceland, Reykjavik, Iceland; 7 Department of Epidemiology, University of Washington, Seattle, WA, USA; 8 Nuffield Department of Medicine, University of Oxford, Oxford, UK

**Keywords:** Pediatrics, Patient characteristics, Outcome, Proteomics

## Abstract

We aimed to establish if enteric permeability was associated with similar biological processes in children recovering from hospitalization and relatively healthy children in the community. Extreme gradient boosted models predicting the lactulose-rhamnose ratio (LRR), a biomarker of enteric permeability, using 7,500 plasma proteins and 34 fecal biomarkers of enteric infection among 89 hospitalized and 60 community children aged 2–23 months were built. The R^2^ values were calculated in test sets. The models performed better among community children (R^2^: 0.27 [min-max: 0.19, 0.53]) than hospitalized children (R^2^: 0.07 [min-max: 0.03, 0.11]). In the community, LRR was associated with biomarkers of humoral antimicrobial and cellular lipopolysaccharide responses and inversely associated with anti-inflammatory and innate immunological responses. Among hospitalized children, the selected biomarkers had few shared functions. This suggests enteric permeability among community children was associated with a host response to pathogens, but this association was not observed among hospitalized children.

## Highlights

• Proteomic models predicted gut permeability better in community than hospital groups
• In the community, enteric permeability was associated with a response to pathogens
• Community biomarkers were also associated with known gut permeability risk factors

## Graphical abstract

**Figure:**
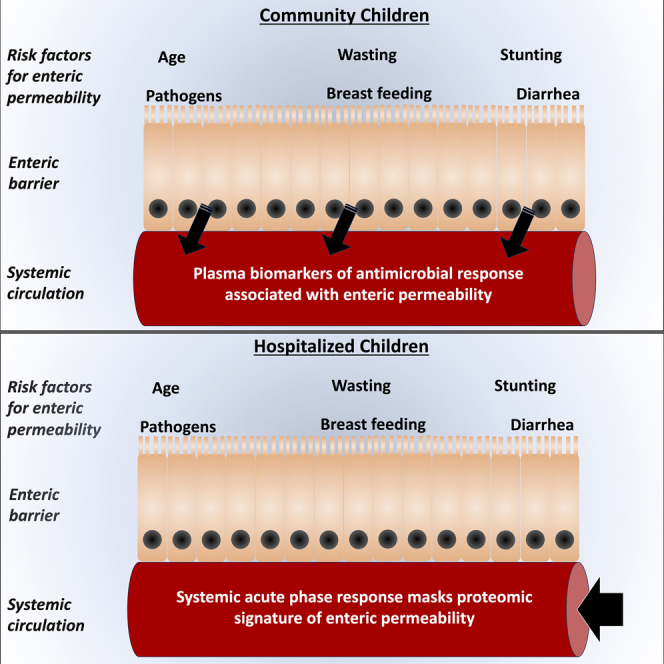

## Introduction

Undernutrition contributes to 45% of all deaths in children under five years of age^1^ and is associated with increased risk of multiple lifelong morbidities.^1^^,^^2^^,^^3^ A combination of social and biological risk factors contributes to undernutrition in many low- and middle-income countries (LMICs). One of the common biological conditions predisposing children to undernutrition is enteric dysfunction (ED), an enteropathy that may be due to frequent exposure to contaminated environments, recurrent enteric infections, micronutrient deficiencies, and early cessation of breastfeeding.^4^^,^^5^ Hallmarks of ED include increased intestinal permeability and a loss of villous structure with decreased absorptive surface area.^3^^,^^4^ Despite the importance of ED to child health outcomes, little is known about the mechanisms that link clinical and demographic risk factors for ED to its enteric pathology and associated morbidity.

Most data evaluating ED in LMIC settings have focused on environmental ED (EED) among asymptomatic community-based children and often find EED to be associated with impaired childhood growth and development.^6^^,^^7^^,^^8^^,^^9^^,^^10^^,^^11^^,^^12^ However, it is unclear if the mechanisms linking ED to adverse outcomes among apparently healthy children in the community also effect children recovering from acute illnesses. Dual sugar testing, including the lactulose-rhamnose ratio (LRR), is a dynamic assessment of enteric permeability that can provide a measure of one aspect of ED.^13^^,^^14^ When LRR is combined with a broad panel of plasma proteins through advanced computational approaches, it may offer insights into the biology of ED among children in the community and in hospital.

We described the biological correlates of ED in apparently healthy children living in communities in Migori (Kenya) and Karachi (Pakistan) and children from the same communities at discharge from hospital following an acute illness. We combine clinical data from the Childhood Acute Illness and Nutrition (CHAIN) Cohort with LRR results collected on a subset of CHAIN children, a proteomic panel of 7,500 plasma biomarkers covering a broad range of biological processes, and a quantitative polymerase chain reaction for 34 enteric pathogens detected on rectal swabs.^15^^,^^16^ Using supervised machine learning we identify plasma proteins associated with the LRR among the community and hospitalized children and we describe the associations between the identified plasma proteins, enteric pathogens, and known clinical and demographic risk factors for increased enteric permeability.

## Results

Of 155 children selected for plasma proteomics analysis, six did not have enteric pathogen data available. Therefore, 60 community children and 89 hospitalized children were included in this analysis (Table 1). The community children were more likely to be from Karachi, breastfed, and HIV unexposed. The hospitalized children had a higher prevalence of wasting, recent diarrhea, and recent antibiotic exposure. Among the hospitalized children, 86 (97%) had received antibiotics during their hospitalization, 36 (40%) had diarrhea at admission or during the hospitalization, 39 (44%) were diagnosed with pneumonia, 26 (29%) were diagnosed with malaria, and six (7%) were diagnosed with sepsis.

**Table 1:** Characteristics of participants included in the machine learning analysis

	Community (N: 60)		Hospital (N: 89)	
Child	N	(%)	n	(%)
Site				
Migori	34	(56.7)	73	(82.0)
Karachi	26	(43.3)	16	(18.0)
Age				
<6 months	16	(26.6)	26	(29.2)
6–12 months	17	(28.3)	28	(31.5)
≥12 months	27	(45.0)	35	(39.3)
Female	26	(43.3)	34	(38.2)
Breastfeeding				
Any current	45	(75.0)	56	(62.3)
Currently exclusive	36	(60.0)	25	(28.1)
Recent antibiotics	10	(16.6)	86	(96.6)
Recent diarrhea	0	(0.0)	36	(40.4)
Stunted^a^	18	(30.0)	32	(36.0)
Wasted^b^	10	(16.6)	38	(42.7)
HIV status				
Unexposed	54	(90.0)	64	(71.9)
Exposed uninfected	6	(10.0)	18	(20.2)
Infected	0	(0.0)	7	(7.9)
Enteric infection				
Cryptosporidium	7	(11.7)	11	(12.4)
ETEC	17	(28.3)	12	(13.5)
Giardia	12	(20.0)	9	(10.2)
Dehydrating pathogens	3	(5.0)	11	(12.4)
Enteroinvasive pathogens	24	(40.0)	60	(67.4)
Caregiver				
Education^c^				
None	14	(23.3)	10	(11.2)
≤Primary	31	(51.7)	53	(59.6)
>Primary	13	(21.7)	26	(29.2)
Body mass index^c^				
Underweight	1	(1.7)	10	(11.2)
Normal	38	(63.3)	65	(73.0)
Overweight/Obese	20	(33.3)	12	(13.5)
Household				
Improved water source^a^	46	(76.7)	62	(69.7)
Improved toilet^a^	33	(55.0)	41	(46.1)
Food insecurity				
Low	25	(41.7)	30	(33.7)
Moderate	28	(46.7)	39	(43.8)
High	7	(11.7)	20	(22.5)

a Defined by WHO criteria (height-for-age z-score <-2).

b Defined by WHO criteria (weight-for-height z-score <−2, or midupper arm circumference <12.5cm if > 6 months old, or edema).

c Missing data: Caregiver education – 2 community; Caregiver BMI – 1 community, 2 hospital.

In the training sets, the community model (root mean squared error [RMSE]: 0.78 [min-max: 0.72, 0.84], R^2^: 0.18 [min-max: 0.09, 0.34]) had better predictive performance than the hospitalized model (RMSE: 1.02 [min-max: 0.99, 1.04], R^2^: 0.04 [min-max: 0.02, 0.07]). The community model (RMSE: 0.67 [min-max: 0.57, 0.69], R^2^: 0.27 [min-max: 0.19, 0.53]) also had better predictive performance than the hospital model (RMSE:1.05 [min-max: 0.99, 1.08], R^2^: 0.07 [min-max: 0.03, 0.11]) in their respective test sets.

### Community model

#### Permeability biomarkers

**Figure 1:**
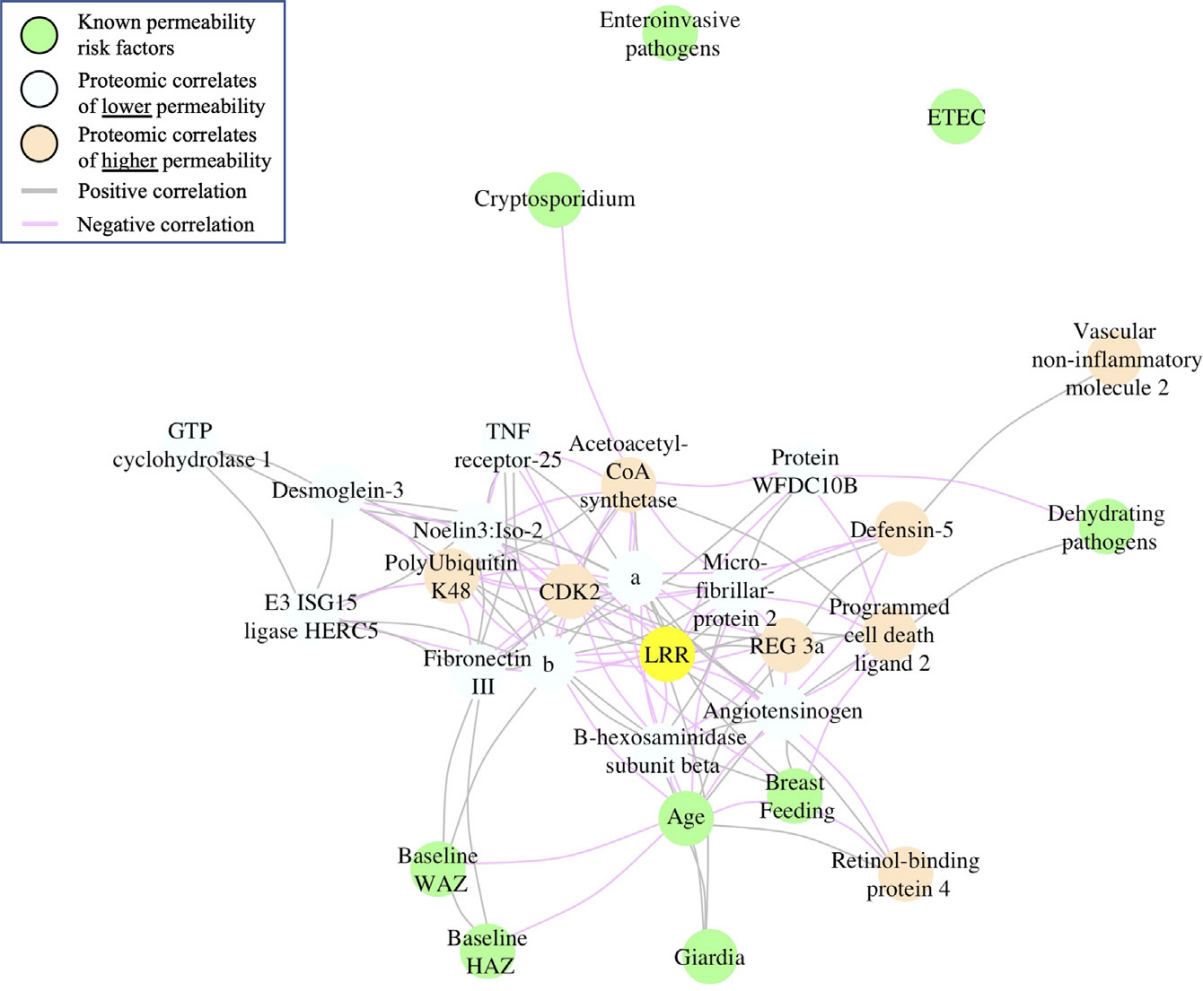
Correlation network of plasma proteins, lactulose-rhamnose ratio, and known enteric permeability risk factor in the community group LRR – lactulose-rhamnose ratio. a, 1-phosphatidylinositol 4,5-bisphosphate phosphodiesterase; b, Proteasome subunit beta type-4.

Eight proteins in the community model were correlated with higher LRR (Table 2, Figure 1). Three of these proteins (regenerating islet-derived protein 3-alpha, defensin-5, programmed cell death 1 ligand 2) are positive regulators of the immune system that contributed to gene ontology (GO)-term biological functions for *humoral antimicrobial response*, *the humoral antimicrobial response mediated by antimicrobial peptide*, and *the cellular response to lipopolysaccharide* (Table S1).

**Table 2:** Plasma proteins associated with lactulose-rhamnose ratio, their gene ontology biological function, and their correlation with known risk factors for enteric permeability

	Community	Model	Hospitalized	Model
Training performance [min, max]	RMSE 0.78 [0.72, 0. 84],	R^2^ 0.18 [0.09, 0.34]	RMSE 1.02 [0.99, 1.04],	R^2^ 0.04 [0.02, 0.07]
Test performance [min, max]	RMSE 0.67 [0.57, 0.69],	R^2^ 0.27 [0.19, 0.53]	RMSE 1.05 [0.99, 1.08],	R^2^ 0.07 [0.03, 0.11]
	Biomarkers of Permeability	Biomarkers of Integrity	Biomarkers of Permeability	Biomarkers of Integrity
Selected proteins	Regenerating islet-derived protein 3a	1-phosphatidylinositol 4,5-bisphosphate phosphodiesterase	R-spondin-3	Macrophage colony stimulating factor 1
	Defensin-5	Proteasome subunit beta type-4	Myocyte-specific enhancer factor 2D	Ephrin-B3:extracellular domain
	Programmed cell death 1 ligand 2	E3 ISG15 protein ligase HERC	Glial fibrillary acidic protein	Vimentin
	Vascular non-inflammatory molecule 2	Protein WFDC10B	Uncharacterized protein C7orf24	High affinity immunoglobulin gamma Fc receptor I
	Retinol-binding protein-4	Tumor necrosis factor receptor superfamily member-25	Guanine nucleotide exchange factor DBS	Receptor-interacting serine/-threonine-protein kinase 2
	Acetoacetyl-CoA synthetase	GTP cyclohydrolase-1	Bifunctional arginine demethylase	Putative KHDC1-like protein
	Cyclin-dependent kinase-2	Angiotensinogen		Bombesin receptor-activated protein C6orf89
	UB2D1/PolyUbiquitin K48	Noelin-3:isoform-2 n-term		Secernin-3
		Desmoglein-3		Tryptase gamma
		Fibronectin type III domain-containing protein-8		Butyrophilin subfamily 2 member A2
		Microfibrillar-associated protein-2		Cyclin-dependent kinase 2:cyclin-A2
		Beta-hexosaminidase subunit beta		Chitinase-3-like protein 2
				Coenzyme Q-binding protein COQ10 homolog A, mitochondrial
				Protein MB21D2
Shared gene ontology biological functions	Humoral antimicrobial response mediated by antimicrobial peptide	Negative regulation of inflammatory response to antigens	Sprouting angiogenesis	Innate immune response
	Antimicrobial humoral response	IgE receptor signaling	Positive regulation of transcription by RNA polymerase II	Inflammatory response
	Cellular response to lipopolysaccharide	T cell receptor signaling		Adaptive immune response
	Positive regulation of cell proliferation	Innate immune response		T cell receptor signaling pathway
	Positive regulation of insulin secretion	TNF-mediated signaling		Cellular response to muramyl dipeptide
	Response to ethanol	Regulation of blood pressure		Cytokine-mediated signaling
	Anaphase promoting complex-dependent catabolic process	Viral process		Positive regulation of cell proliferation
		Signal transduction		Signal transduction
		Protein polyubiquitination		
Risk factors of increased biomarker expression	Older age	Breastfeeding	Older age	Breastfeeding
	Dehydrating enteric pathogen	Giardia		
Risk factors of decreased biomarker expression	Cryptosporidium	Older age	–	–
	Giardia	Dehydrating enteric pathogen		

Min, minimum; Max, maximum; RMSE, root mean squared error.

Two of the “permeability biomarkers” (cyclin-dependent kinase-2, UB2D1/PolyUbiquitin K48) in the community model contributed to GO terms for *positive regulation of cell proliferation* and *anaphase promoting complex dependent catabolic process*. Another two “permeability biomarkers” (retinol-binding protein-4, acetoacetyl-coenzyme A [CoA] synthetase) are associated with GO terms for *response to ethanol* and the *positive regulation of insulin secretion*. The final “permeability biomarker” (vascular non-inflammatory molecule 2) did not share a GO-term biological function with any of the other biomarkers but is thought to be involved in thymus homing of bone marrow cell and may regulate migration of neutrophils.

These “permeability biomarkers” were associated with known risk factors for elevated LRR (Figures 1 and S1). The detection of a dehydrating enteric pathogen was correlated with higher levels of programmed cell death 1 ligand 2 (correlation [corr] = 0.3, 95% confidence interval [CI]: 0.1, 0.5, p = 0.020). Older age was also moderately correlated with higher programmed cell death 1 ligand 2 (corr = 0.4, 95% CI: 0.2, 0.5, p = 0.001). Conversely, *Cryptosporidium* detection was correlated with lower acetoacetyl-CoA synthetase (corr = −0.4, 95% CI: −0.1, −0.6, p = 0.003), while *Giardia* was correlated with lower regenerating islet-derived protein 3-alpha (corr = −0.3, 95% CI: −0.0, −0.5, p = 0.023), defensin-5 (corr = −0.3, 95% CI: −0.0, −0.5, p = 0.040), and retinol-binding protein-4 (corr = −0.3, 95% CI: −0.0, −0.5, p = 0.037). Finally, breastfeeding was correlated with lower regenerating islet-derived protein 3-alpha (corr = −0.3, 95% CI: −0.0, −0.5, p = 0 · 046), programmed cell death 1 ligand 2 (corr = −0.3, 95% CI: −0.1, −0.6, p = 0.007), and retinol-binding protein-4 (corr = −0.3, 95% CI: −0.1, −0.5, p = 0.001).

#### *Integrity* biomarkers

Twelve proteins in the community model were correlated with lower LRR. Five of these biomarkers had functions relating to the regulation the immune response, including those with GO terms for *the negative regulation of inflammation in response to antigens*, *IgE receptor signaling*, *T cell receptor signaling*, *the innate immune response*, and *TNF-mediated signaling*. These proteins were 1-phosphatidylinositol 4,5-bisphosphate phosphodiesterase; proteasome subunit beta type-4; E3 ISG15 protein ligase HECT And RLD Domain Containing E3 Ubiquitin Protein Ligase 5 (HERC5); protein WAP Four-Disulfide Core Domain 10B (WFDC10B); and tumor necrosis factor receptor superfamily member-25. These proteins also contributed to the GO-terms functions for *signal transduction*, *protein polyubiquitination*, and *viral processes*.

Guanosine triphosphate (GTP) cyclohydrolase-1 and angiotensinogen were also identified as “integrity biomarkers”. These proteins play antagonistic roles in *the regulation of blood pressure* and are known to be inversely associated with age. The remaining “integrity biomarkers” in the community model were noelin-3:isoform-2 n-term, desmoglein-3, beta-hexosaminidase subunit beta, fibronectin type III domain-containing protein-8, and microfibrillar-associated protein-2. However, these proteins did not share any GO-terms biological functions with other biomarkers.

Known risk factors for enteric permeability were correlated with some of the “integrity biomarkers”. Detection of a pathogen in the dehydrating group was correlated with lower levels of protein WFDC10B (corr = −0.3, 95% CI: −0.1,-0.5, p = 0.012). Older age demonstrated multiple negative correlations with integrity biomarkers: beta-hexosaminidase subunit beta (corr = −0.3, 95% CI: −0.1, −0.5, p = 0.010), 1-phosphatidylinositol 4,5-bisphosphate phosphodiesterase (corr = −0.3, 95% CI: −0.1, −0.6, p = 0.006), proteasome subunit beta type-4 (corr = −0.3, 95% CI: −0.1, −0.6, p = 0.007), microfibrillar-associated protein-2 (corr = −0.3, 95% CI: −0.1, −0.5, p = 0.010), and angiotensinogen (corr = −0.6, 95% CI: −0.3, −0.7, p < 0.001).

*Giardia* detection was correlated with beta-hexosaminidase subunit beta (corr = 0.4) and microfibrillar-associated protein-2 (corr = 0.3, 95% CI: 0.1, 0.6, p = 0.005). Finally, breastfeeding was correlated with higher 1-phosphatidylinositol 4,5-bisphosphate phosphodiesterase (corr = 0.3, 95% CI: 0.1, 0.5, p = 0.010), proteasome subunit beta type-4 (corr = 0.3, 95% CI: 0.1, 0.6, p = 0.002), noelin-3:isoform-2 n-term (corr = 0.4, 95% CI: 0.0, 0.5, p = 0.024), and angiotensinogen (corr = 0.4, 95% CI: 0.1, 0.6, p = 0 · 004), while both height-for-age z-score (HAZ, corr = 0.4, 95% CI: 0.1, 0.6, p = 0.001) and weight-for-age z-score (WAZ, corr = 0.4, 95% CI: 0.1, 0.6, p = 0.001) were correlated with higher fibronectin type III domain-containing protein-8; WAZ was also correlated with beta-hexosaminidase subunit beta (corr = 0.3, 95% CI: 0.0, 0.5, p = 0.051).

### Hospital model

In addition to having worse performance, the hospital model’s selected proteins had fewer shared functions and fewer correlations with known risk factors for enteric permeability (Figures S2 and S3).

#### Permeability biomarkers

Among six “permeability biomarkers” in the hospitalized model, the only GO-term functions shared by two or more proteins were *sprouting angiogenesis* and *positive regulation of transcription by RNA polymerase II* (Table S2) These permeability-associated proteins were R-spondin-3, myocyte-specific enhancer factor 2d, glial fibrillary acidic protein, uncharacterized protein C7orf24, guanine nucleotide exchange factor DBS, and bifunctional arginine demethylase.

Increased age was correlated with higher guanine nucleotide exchange factor DBS (corr = 0.4, 95% CI: 0.1, 0.5, p < 0.001). Detection of a pathogen in the enteroinvasive group was correlated with lower levels of the cell growth biomarker myocyte-specific enhancer factor 2d (corr = −0.3, 95% CI: −0.7, 0.2, p = 0.235). No other known risk factors for enteric permeability were correlated with “permeability biomarkers” in the hospital model.

#### *Integrity* biomarkers

Among the 14 biomarkers associated with lower LRR in the hospital model, five proteins reflect immunological regulation pathways, including GO terms for *innate immune response*, *inflammatory response*, *adaptive immune response*, *T cell receptor signaling*, *a cellular response to muramyl dipeptide*, and *cytokine-mediated signaling*. These proteins were macrophage colony stimulating factor 1, butyrophilin subfamily 2 member A2, ephrin-B3:extracellular domain, vimentin, and receptor-interacting serine/threonine-protein kinase-2. High affinity immunoglobulin gamma Fc receptor I was also selected as an “integrity biomarker” in the hospital model and contributed a GO term for *signal transduction* with receptor-interacting serine/threonine-protein kinase-2 and cyclin-dependent kinase-2:cyclin-A2. A final GO-term biological function for *positive regulation of cell proliferation* was contributed by macrophage colony stimulating factor 1 and cyclin-dependent kinase 2:cyclin-A2.

The other “integrity biomarkers” in the hospital model were putative KH homology domain-containing protein 1 (KHDC1)-like protein, bombesin receptor-activated protein C6orf89, secerning-3. tryptase gamma, coenzyme Q-binding protein COQ10 homolog A, chitinase-3-like protein 2, and protein (MB21D2). However these proteins did not contribute to GO-term functions.

Breastfeeding was the only known permeability risk factor which was correlated with an “integrity biomarker” (vimentin, corr = 0.4, 95% CI: 0.1, 0.5, p < 0.001) in the hospitalized model.

## Discussion

Using panels of 7,500 plasma proteins we found models predicting the LRR among children in the community to be more accurate than predictions made among similarly aged hospitalized children recovering from acute illnesses. In LMICs, enteric pathogens are thought to drive leukocyte invasion into the gut wall and consequently lead to the development of ED.^13^^,^^17^ The biomarkers associated with permeability in the community model shared biological functions related to humoral antimicrobial immunity and response to lipopolysaccharide which is an outer membrane component of gram-negative bacteria. These “permeability biomarkers” included regenerating islet-derived protein 3-alpha and defensin-5 which are indicative of a host response to infection and are associated with the enteric system. In addition to the shared GO terms, several of the permeability biomarkers are known to be important in leukocyte proliferation and migration into peripheral tissues such as the gut, including programmed cell death 1 ligand 2 (T cell proliferation) and vascular non-inflammatory molecule 2 (neutrophil migration). The identified “permeability biomarkers” in the community were also correlated with known demographic and clinical risk factors for enteric permeability, including dehydrating enteric pathogens, older age, and breastfeeding cessation. Conversely, lower LRR in the community model was associated with biomarkers of a suppressed response to antigens and the innate immune response. Collectively the results of our community model support the hypothesis that a host response to enteric pathogens contributes to the widespread ED observed among children in LMIC communities.

Current breastfeeding was correlated with lower biomarkers of “permeability” in the community and higher biomarkers of “integrity” in the hospitalized model. These biomarkers suggested that breastfeeding was associated with the negative regulation of antigenic responses and lower levels of pro-inflammatory proteins like regenerating islet-derived protein 3-alpha and programmed cell death 1 ligand 2. Other research has also found that breastfeeding was an important factor in reducing ED among young children.^11^^,^^18^^,^^19^ The children in this study were 2–23 months of age, which includes a period of childhood when the transition from exclusive breastfeeding to family foods signifies the waning of passive immunity provided by breast milk, and an increased exposure to enteric pathogens. While we cannot draw causal conclusions from this analysis, our findings are consistent with the hypothesis that breastfeeding improved intestinal barrier integrity by downregulating inflammatory pathways and limiting pathogen exposure.

The community model explained 27% of the variability in the LRR while the hospitalized model explained only 10%. Previous analysis of this dataset found that LRR was associated with systemic inflammation among the community cohort but not the hospitalized children.^20^ Hospitalizations in this age group are predominantly caused by acute infections which will result in a broad proteomic disturbance caused by the acute phase response. This proteomic disruption may make it very difficult to detect enteropathy-associated plasma biomarkers, which are often themselves inflammatory proteins, and this may explain the poor model performance among hospitalized children. However, neither model was highly accurate. The LRR test is known to produce heterogeneous results, influenced by factors such as gastric emptying, intestinal motility, renal clearance, and frequency of urinary voiding, making it a challenging target for predictive modeling. Additionally, increased enteric permeability is a pathology of the enteric mucosa, and plasma biomarkers may only capture downstream consequences of mucosal disruption, such as translocation and the immune response.

We found enteric permeability to be correlated with a cluster of plasma proteins indicative of an inflammatory response to pathogens among children in the community. We were also able to correlate these biomarkers with some known risk factors for gut permeability. These data are consistent with the hypothesis that exposure to enteric pathogens may be a prevalent risk factor, and breastfeeding may be a critical protective factor, for ED among children in sub-Saharan African and south Asian communities. However, a similar model among hospitalized children recovering from acute illness performed substantially less well, underscoring the challenges of studying enteric function among a group of children recovering from acute illness from multiple causes.

### Limitations of the study

This analysis leverages rigorously conducted LRR testing, state-of-the-art plasma proteomics, and highly sensitive quantitative PCR analysis. However, the analytic methods applied are designed for hypothesis generation and not causal modeling. Our study enrolled a highly heterogeneous population, with and without acute illnesses, and from two different countries. While this diversity may serve to strengthen the generalizability of the machine learning models, it can also complicate interpretation. We also note that more community children were recruited from Karachi, which may have introduced a bias if the model worked better in Karachi. We also were not able to include all the recruited children in the model as a small number of rectal swab samples were not available for analysis, and approximately 10% of children had failed LRR test. This may have introduced a degree of selection bias. Our community sampling strategy was pseudorandom and required clinic attendance, which may mean our community group is not representative of the community as a whole. However, we do note that our community results are well aligned with other community studies of ED. Finally, some of the proteins our model associated with the LRR have poorly understood biological functions; there may be unknown biological functions that link these proteins and are important ED mechanism.

## STAR★Methods

### Key resources table

**Table:** 

REAGENT or RESOURCE	SOURCE	IDENTIFIER
**Biological samples**		

	The Childhood Acute Illness & Nutrition Network Cohort Study	

**Critical commercial assays**		

The Somascan assay	Somalogic, Boulder, CO	

**Deposited data**		

	CHAIN Network data available at https://dataverse.harvard.edu/dataset.xhtml;jsessionid=7f7a2798b3195f9cd5a1cb4152d1?persistentId=doi%3A10.7910%2FDVN%2F5H5X0P&version=&q=&fileTypeGroupFacet=&fileAccess=Public&fileSortField=size CHAIN Lactulose Rhamnose substudy available at https://doi.org/10.5061/dryad.9zw3r22kd	

### Resource availability

#### Lead contact

Further information and requests for resources and reagents should be directed to and will be fulfilled by the lead contact, Kirkby Tickell (kirkbt@uw.edu).

#### Materials availability

This study did not generate new unique reagents.

### Experimental model and study participant details

This study included acutely ill children aged 2−23 months enrolled in the CHAIN study.^15^^,^^16^^,^^21^^,^^22^ Two CHAIN sites, Migori County Referral Hospital in western Kenya and Civil Hospital in Karachi, Pakistan, participated in the LRR sub-study. No gender or sex bias was introduced in the enrolling strategy for these children, but the effect of sex in this dataset has been previously explored.^20^ Information on the sample size is provided in the method details section below, and the Result. Ethical approval was obtained from the University of Washington, University of Oxford, the Kenya Medical Research Institute, and Aga Khan ethical review boards.

### Method details

#### Parent study enrollment and follow-up

The CHAIN Cohort was a prospective study which enrolled acutely ill children aged 2−23 months across nine sites in six countries in Africa and South Asia at admission to hospital.^15^^,^^16^^,^^21^^,^^22^ Children were enrolled across a range of rural and urban environments and differing malaria and HIV endemicities. Two CHAIN sites, Migori County Referral Hospital in western Kenya and Civil Hospital in Karachi, Pakistan, participated in the LRR sub-study.

CHAIN participants were stratified by child mid-upper arm circumference (MUAC), with sites aiming to recruit two children with very low MUAC (<11.5 cm if older than 5 months, otherwise <11 cm) or bipedal edema, and two with moderately low MUAC (≥11.5 cm but <12.5 cm if older than 5 months, otherwise ≥11 cm but <12.0 cm) for each child with a normal MUAC (≥12.5 cm if older than 5 months, otherwise ≥12.0 cm). Detailed clinical, anthropometric, and sociodemographic data were collected at admission and discharge in addition to blood and rectal swab samples. Daily clinical observations and management were recorded on standardized case report forms during admission. Home environment characteristics were assessed at home visits at discharge.

The CHAIN study also recruited community reference participants from households near the hospitalized children’s homes, using a pseudo-random selection method (3^rd^ house to the north of the enrolled child’s house). Community children were recruited in the same age bracket as the index hospitalized child (<6 months, 6–11 months, 12–23 months) if they had no history of acute illness in the 14 days prior, and if their caregiver consented to participation, and if they had not previously been included in the study. A community recruitment was attempted for every hospitalized child who was discharged. If a child was not found the fieldworker would continue to the next house in the same direction. Demographics, medical history and examination, and anthropometry data and sample collection were obtained from community children using the same methods as in the hospitalized children.

#### Sub-study enrollment

Children enrolled in CHAIN at the Civil Hospital Karachi (March 2018-September 2019) and the Migori County Referral Hospital (December 2017-October 2019) sites, including the hospitalized and community groups, were eligible for inclusion in this sub-study when they were determined to be medically stable (no respiratory distress, not requiring supplemental oxygen, and nutritional intake was by oral route). The first three eligible children each week were selected for participation to facilitate accurate implementation of the LRR test. Additional informed consent was obtained prior to inclusion in this sub-study. Both hospitalized and community children with diarrhea on the day of the LRR test were excluded, as lactulose may exacerbate diarrhea.

#### The lactulose-rhamnose test

Dual sugar tests employ oral administration of a smaller sugar (rhamnose) that can readily cross a healthy enteric barrier into the circulation and a larger sugar (lactulose) which can only permeate (to any appreciable extent) a disrupted barrier. Both sugars are metabolically inert and excreted in urine. The LRR test was conducted in the morning and caregivers were asked to fast (food, drink, and breastmilk) their child for 1 h. A 10 mL oral solution containing 1500 mg lactulose and 300 mg L-rhamnose was administered at the end of the fasting hour and a new urine bag attached. Urine passed in the first 20 min after sugar administration was discarded and all urine passed during the subsequent 2 h was collected. The caregiver was encouraged to provide breastmilk or water to the child after administration of the sugar solution. Any stool contamination of the urine bag, urine leakage, or failure to void in the 2-h post-administration period were considered a test failure. Failed tests were repeated after 24-h if caregivers were willing.

Urine samples from each time period (20–80 min and 80–140 min post-sugar administration) were aliquoted into 100 μL cryovials and stored at −80^o^c within 1 h of collection. These aliquots were shipped to the Mayo Clinic (Rochester, Minnesota) for high-performance liquid chromatography mass spectrometry. Percentage of lactulose and rhamnose recovery was calculated for each post-administration time period, as was LRR. The cumulative LRR encompassing both time periods was calculated by deriving a mean concentration of lactulose and rhamnose weighted to the volume of urine passed in each post-administration period. In keeping with previous LRR analyses, failure to detect rhamnose in the post-administration sample was classified as a test failure.^10^^,^^20^

#### Plasma biomarkers

Blood samples, were processed in site laboratories within 1 h of collection. Samples were spun in a refrigerated centrifuge into plasma and serum, stored and shipped at −80°C. We analyzed plasma samples from a subset of children who had successfully participated in the LRR sub-study who had both plasma and rectal swab samples available. We were able to fund the analysis of 155 samples, and selected 95 children from the hospitalized group and 60 from the community group. The hospitalized children were selected at a higher rate to allow for previously published analysis of their growth data. Children with failed LRR tests (n = 24 of 245 tests) were not eligible biomarker analysis. The proteomic approach was implemented by Somalogic, Inc using an aptamer-based technology, which assesses the concentration of 7,500 proteins.^15^^,^^23^^,^^24^

#### Quantitative PCR of enteric pathogens

Copan flocked rectal swabs were collected and stored at −80°C within in 1-h of collection. TACman quantitative PCR for 32 enteric pathogens was conducted on these rectal swabs.^15^ Cycle threshold values under 30 were considered positive detections. These data were aggregated into variables for detection of any pathogen within specific groups at either admission or discharge. The pathogens were grouped in accordance with Kosek et al.^11^: dehydrating pathogens (rotavirus, adenovirus, norovirus, and astrovirus), and enteroinvasive pathogens (*Campylobacter, Shigella/Enteroinvasive Escherichia coli* (*E.coli*)*, Salmonella, Plesiomonas, Yersinia*, enteroaggregative *E.coli*, enteropathogenic *E.coli,* and *Aeromonas*). Enterotoxic *E.coli, Cryptosporidium* and *Giardia* were analyzed independently as their pathogenic mechanisms are not thought to cluster well with the other groups.

### Quantification and statistical analysis

Hospitalized and community groups were assessed separately, because previous analysis of this dataset suggested that plasma biomarkers from the community did not generalize to the hospital cohort.^20^ The LRR as a continuous variable was the outcome of interest for predictive models, and the qPCR groups as outlined above and the full panel of proteins were available as potential predictors. All data were standardized (i.e., z-scores were creates), including the outcome. The aptamer-based proteomics do not naturally fall into clinically interpretable units, making standardization the most logical means of interpreting these variables in models (i.e., one unit = one standard deviation change). Missing values were imputed using a K-nearest neighbor method.^25^

Available data were split into training (75%) and test sets (25%). We choose a 75:25 split heuristically. There is no agreed upon method for choosing the training/test ratio, although there is a weak consensus that large dataset can probably use ratios of 80:20 or even 90:10 effectively. Small dataset tend to choose lower ratios of 70:30 or 75:25. Machine learning projects tend not to vary the training/test ratios, as choosing between the ratios could compromise the validity of the test set. Extreme gradient boosted (XGBoost) models were tuned in the training sets using 10-fold cross validation. The final tuned models were applied to the test set, and the root-mean-square error (RMSE) and R^2^ statistics calculated. A “leave one out” algorithm was used to estimate the minimum and maximum (min-max) RMSE and R^2^. XGBoost modeling was chosen as this approach is highly flexible, but also contains a penalization term that aims to minimize overfitting which makes it suitable for a wide variety of predictive challenges.^25^

To better understand the variables contributing to the model’s prediction, variable importance (i.e., those variables which most informed the model prediction) was analyzed. Biomarkers associated with increased LRR are described as permeability biomarkers while those correlated with lower LRR are referred to as *integrity biomarkers.* The top 20 most influential variables were matched to their Gene Ontology (GO) term, which list the known biological functions of proteins, in the UniProt database.^26^ To understand the relationships between these predictors Pearson’s correlation coefficient matrices were calculated and displayed using heatmaps and network diagrams. Finally, *a-priori* identified risk factors for increased or decreased permeability were added to the correlation matrices, including weight-for-age *Z* score (WAZ), length-for-age *Z* score (LAZ), age, any current breastfeeding, history of diarrhea at admission or during the hospitalization, and enteric pathogen groups (dehydrating, enteroinvasive, ETEC*, Cryptosporidium,* and *Giardia)*. These risk factors were chosen on the basis of evidence published in other studies among similarly aged children.^11^^,^^18^^,^^19^^,^^27^^,^^28^ Correlation coefficients ≥0.3 were described as correlated, while those <0.30 were considered not correlated.^29^

No children included in this study were missing lactulose rhamnose ratio, quantitative PCR or proteomic data. However, three children had missing data of recent diarrhea, which was assumed to be missing at random and was imputed using a K-nearest neighbor method in R’s Caret package. All other risk factor data was complete.

### Additional resources

CHAIN Cohort registration on clinicaltrials.gov: https://classic.clinicaltrials.gov/ct2/show/NCT03208725.

## Data Availability

• De-identified data have been deposited at Dyrad, https://doi.org/10.5061/dryad.9zw3r22kd. They are publicly available as of the date of publication. Accession numbers are listed in the key resources table. • All original code has been deposited at Dyrad, https://doi.org/10.5061/dryad.9zw3r22kd and is publicly available as of the date of publication. DOIs are listed in the key resources table. • Any additional information required to reanalyze the data reported in this paper is available from the lead contact upon request.
