# Supplementary material for: Plasma proteomic signatures of enteric permeability among hospitalized and community children in Kenya and Pakistan
Source: iScience. 2023 Jul 11;26(8):107294. doi: 10.1016/j.isci.2023.107294 (PMC10405056; doi:10.1016/j.isci.2023.107294)
Supplement: Document S1. Figures S1–S3, Tables S1, and S2 [file mmc1.pdf]

iScience, Volume *26*

Supplemental information

Plasma proteomic signatures of enteric permeability among hospitalized and community children in Kenya and Pakistan

Kirkby D. Tickell, Donna M. Denno, Ali Saleem, Zaubina Kazi, Benson O. Singa, Catherine Achieng, Charles Mutinda, Barbra A. Richardson, Kristjana H. Ásbjörnsdóttir, Stephen E. Hawes, James A. Berkley, and Judd L. Walson

Table S1: Biomarkers from the community model with gene-ontology terms shared by two or more proteins, related to Table 2.

	**Gene ontology terms from biomarkers of permeability**							**Gene ontology terms from biomarkers of integrity**								
	Humoral antimicrobial response mediated by antimicrobial peptide	Antimicrobial humoral response	Cellular response to LPS	Positive regulation of cell proliferation	Response to ethanol	Positive regulation of insulin secretion	Anaphase promoting complex-dependent catabolic process	Negative regulation of inflammatory response to antigens	IgE receptor signaling	T-cell receptor signaling	Innate immune response	TNF-mediated signaling	Viral process	Protein polyubiq-uitination	Regulation of blood pressure	Signal transduct-ion
**Regenerating islet-derived protein 3a**	■	■		■												
**Defensin-5**	■	■	■													
**Programmed cell death 1 ligand 2**			■													
**Vascular non-inflammatory molecule 2**																
**Retinol-binding protein-4**					■	■										
**Acetoacetyl-CoA synthetase**					■	■										
**Cyclin-dependent kinase-2**				■			■									
**UB2D1/PolyUbiquitin K48**				■			■									
**1-phosphatidylinositol 4,5-bisphosphate phosphodiesterase**								■	■	■			■			■
**Proteasome subunit beta type-4**								■	■	■		■	■	■		■
**E3 ISG15 protein ligase HERC**											■			■		
**Protein WFDC10B**											■					
**Tumor necrosis factor receptor superfamily member 25**												■				
**GTP cyclohydrolase-1**															■	
**Angiotensinogen**															■	
**Noelin-3:isoform-2 n-term**																
**Desmoglein-3**																
**Fibronectin type III domain- containing protein-8**																
**Microfibrillar-associated protein-2**																
**Beta-hexosaminidase subunit beta**																

Figure S1: Heat map of the correlations between selected plasma proteins, lactulose-rhamnose ratio and known enteric permeability risk factor in the community group, related to Figure 1.

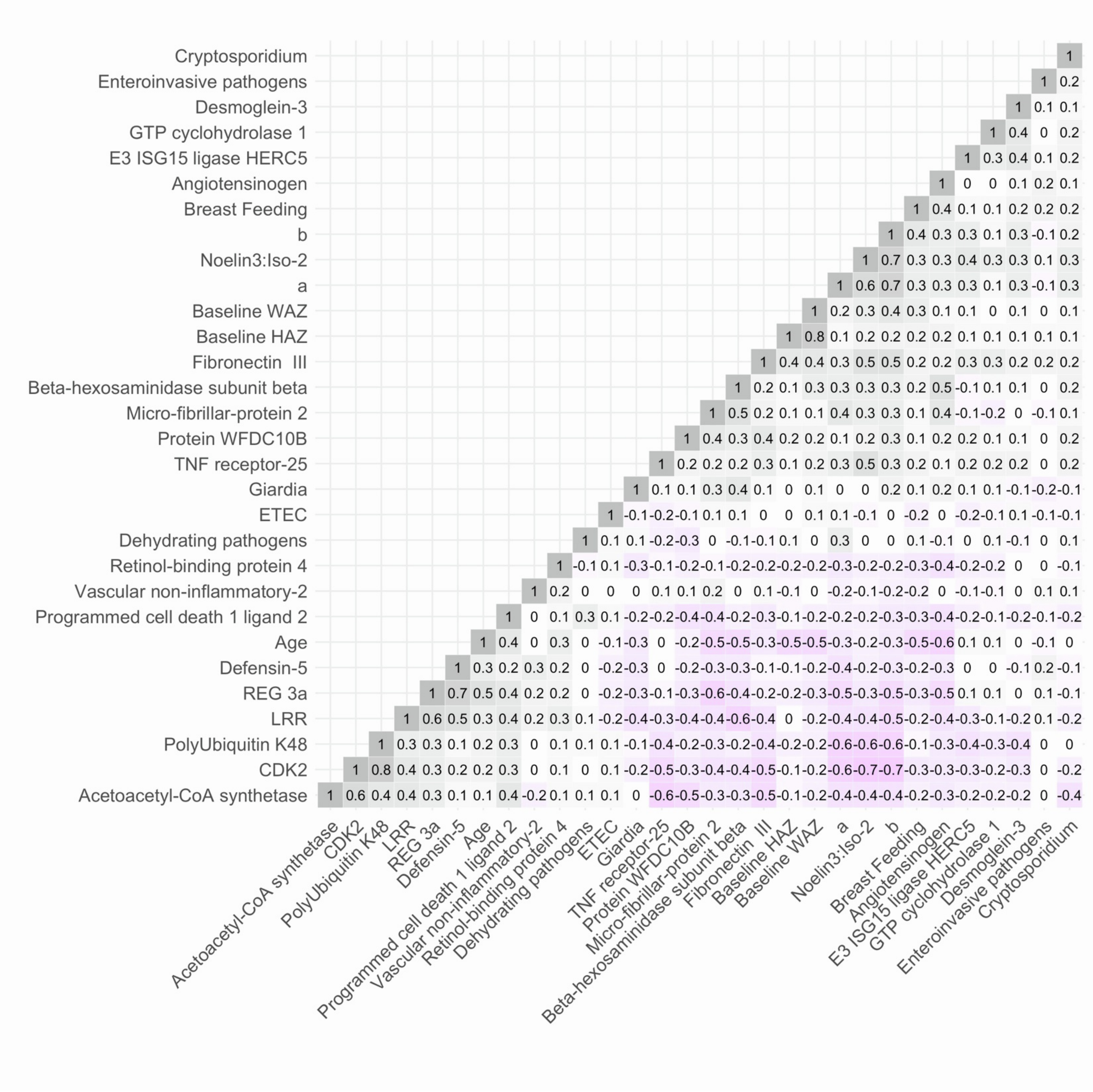

Figure S2: Correlation network of plasma proteins, lactulose-rhamnose ratio and known enteric permeability risk factor in the hospitalized group, related to Figure 1.

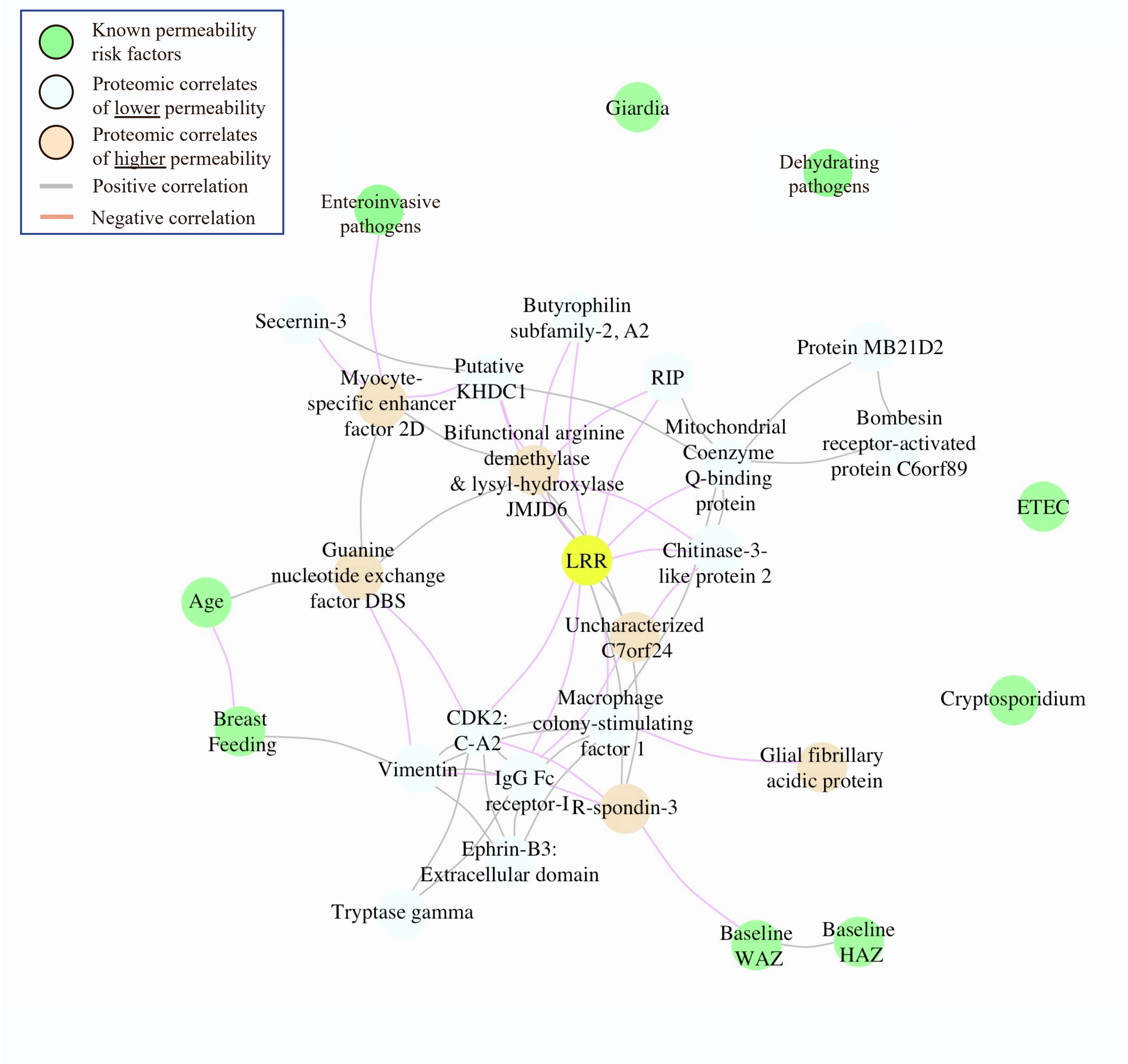

Table S2: Biomarkers from the hospital model with gene-ontology terms shared by two or more proteins, related to Table 2.

	**Gene ontology terms from biomarkers of permeability**		**Gene ontology terms from biomarkers of integrity**							
	Sprouting angiogenesis	Positive regulation of transcription by RNA polymerase II	Innate immune response	Inflammatory response	Adaptive immune response	T-cell receptor signaling pathway	Cellular response to muramyl dipeptide	Cytokine mediated signaling	Positive regulation of cell proliferation	Signal transduction
**Myocyte-specific enhancer factor 2D**		■								
**R-spondin-3**	■									
**Glial fibrillary acidic protein**										
**Guanine nucleotide exchange factor DBS**										
**Bifunctional arginine demethylase**	■	■								
**Uncharacterized protein C7orf24**										
**Macrophage colony-stimulating factor 1**			■	■				■	■	
**Butyrophilin subfamily 2 member A2**					■	■				
**Ephrin-B3:Extracellular domain**										
**Vimentin**			■				■	■		
**High affinity immunoglobulin gamma Fc receptor I**										■
**Receptor-interacting serine/threonine-protein kinase 2**			■	■	■	■	■			■
**Putative KHDC1-like protein**										
**Bombesin receptor-activated protein C6orf89**										
**Secernin-3**										
**Tryptase gamma**										
**Cyclin-dependent kinase 2:Cyclin-A2 complex**									■	■
**Coenzyme Q-binding protein COQ10 homolog A, mitochondrial**										
**Chitinase-3-like protein 2**										
**Protein MB21D2**										

Figure S3: Correlation between plasma proteins, lactulose-rhamnose ratio and known enteric permeability risk factor in the hospitalized group, related to Table 2.

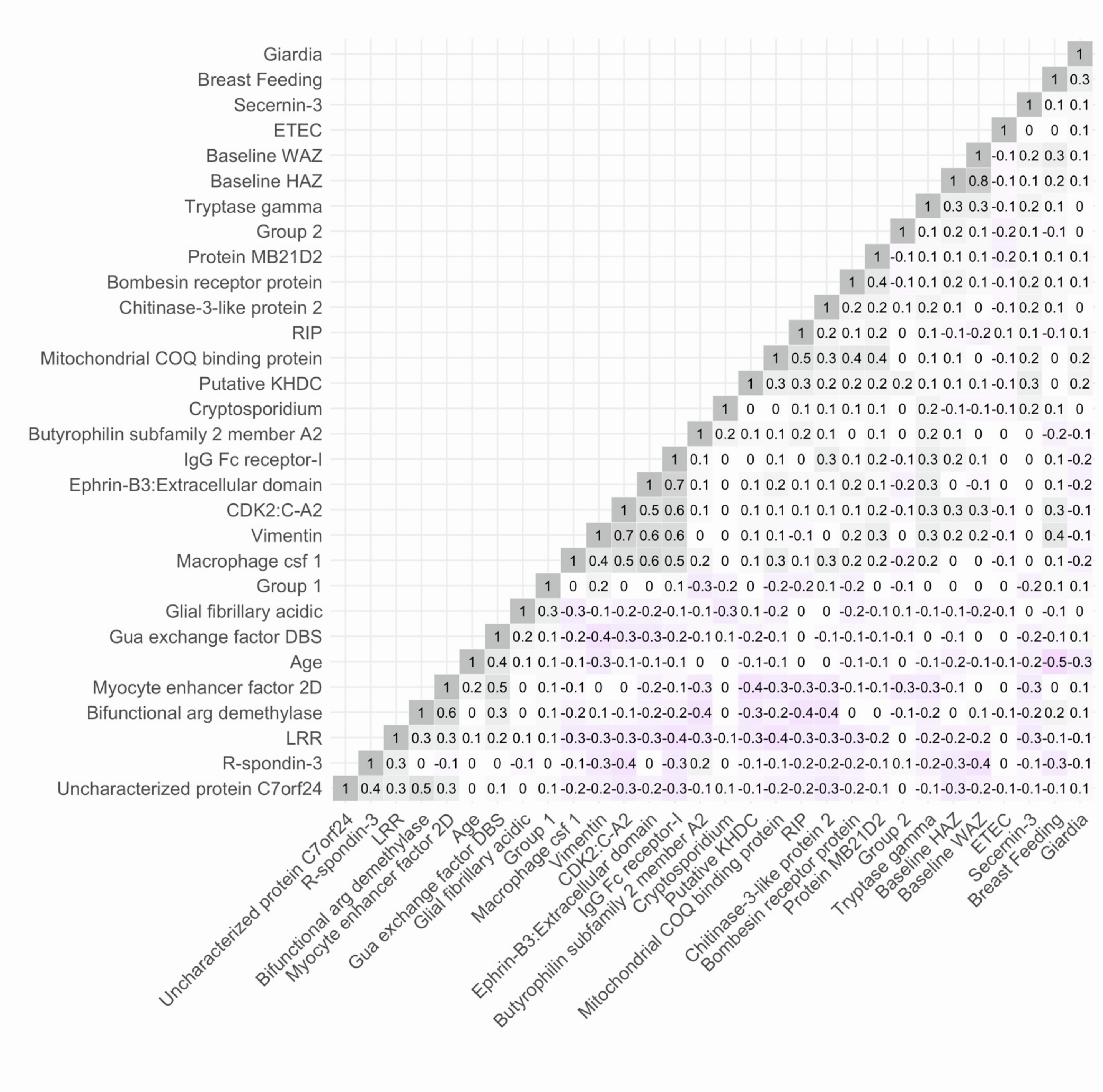

Group 1: Dehydrating enteric pathogens. Group 2: Enteroinvasive pathogens.
